# Development of CuO/Cu_4_(OH)_6_SO_4_ Nanoparticle Mixtures to Optimize the H_2_S Adsorption

**DOI:** 10.1021/acsaenm.3c00575

**Published:** 2024-01-22

**Authors:** Donald Hill, Yubiao Niu, Henry Apsey, Omotoke Olonisakin, Richard E. Palmer, Shirin Alexander

**Affiliations:** † Energy Safety Research Institute (ESRI), Faculty of Science and Engineering, Swansea University, Bay Campus, Fabian Way, Swansea SA1 8EN, U.K.; ‡ Nanomaterials Lab, Faculty of Science and Engineering, Swansea University, Bay Campus, Fabian Way, Swansea SA1 8EN, U.K.

**Keywords:** hydrogen sulfide, sorbent, copper oxide, nanowire, filter, malodor

## Abstract

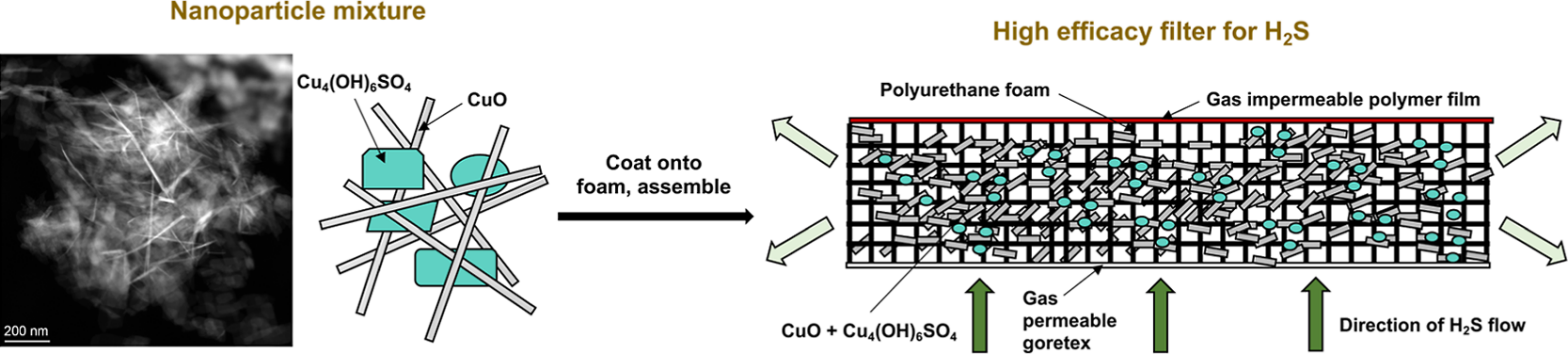

In this paper, we report the H_2_S adsorption behavior of a sorbent composed of mixtures of tenorite (CuO) and brochantite [Cu_4_(OH)_6_SO_4_]. These materials are readily prepared through the addition of NaOH_(aq)_ to CuSO_4(aq)_. They can be loaded onto polymer foams to create effective filters that can remove malodorous H_2_S gas, as evidenced by breakthrough tests. X-ray diffraction shows that the ratio of the two compounds in the mixture can be finely tuned by varying the amount of NaOH_(aq)_ that is added to the reaction mixture. X-ray photoelectron spectroscopy shows that brochantite, like tenorite, has the ability to chemically adsorb H_2_S. Correlation of the H_2_S breakthrough data with scanning transmission electron microscopy measurements shows that the most effective sorbents contain nanoscale needle-like particles. These are likely to be formed largely by the tenorite phase. The samples with the greatest H_2_S adsorption efficacy contained less than 20% tenorite in the mixture, where these particles had the greatest abundance. The application of this sorbent onto porous substrates to create effective filters, along with the synthetic ease of its production, could allow this methodology to find use in a number of areas where H_2_S poses a problem. This could include areas where protective clothing is required to adsorb the gas from environments where there is a high level of H_2_S, for example, in wastewater treatment plants, oil and gas wells, or in the medical sector, where it could be deployed as filter media.

## 1 Introduction

Removal of hydrogen sulfide (H_2_S) from natural and anthropogenic sources has attracted much interest from the industrial and scientific community. Hydrogen sulfide is a colorless, corrosive gas that is toxic to humans at ppm levels, with a distinct “rotten egg” smell.^1−3^ Biologically, it is formed due to bacterial breakdown of organic matter through bacterial desulfhydration of cysteine residues^4^ and as a byproduct during ATP production in sulfate reducing bacteria.^5^ In addition, hydrogen sulfide is also produced bacterially in some of the anaerobic conditions that occur in wastewater treatment^6^ and in oil and gas wells at concentrations where it poses a risk to life.^7^ Because of this, there has been substantial research into methods and materials that can remove hydrogen sulfide from both the gas and aqueous phase.

A large number of sorbents and methods have been developed for the removal of H_2_S that utilize appropriate chemistry to adsorb or react with the molecule from the gas or liquid phase. Successful sorbents have been observed to include materials such as zeolites,^8−10^ metal oxides,^10,11^ porous carbons,^9,10^ and mesoporous silicas.^9,10^ Adsorption has been observed to occur by either physical or chemical means, and its effectiveness is unsurprisingly dependent on material surface area. Sorbents possessing suitable surface chemistry to chemically adsorb H_2_S have been shown to have better performance relative to those relying solely on physical adsorption alone. Chemical adsorption has been observed to occur at the surface of metal oxides, whereby sulfidation occurs, leading to the formation of metal sulfides and sulfate species.^10−12^ Alternatively, H_2_S chemisorption has been observed to occur on materials bearing N-containing moieties, such as activated charcoals bearing pyridinic nitrogens^13^ or those derived from alkyl amine compounds.^14^ Methodologies used to design highly effective sorbents tailor both the surface chemistry and surface area to create materials that possess the greatest number of sites onto which H_2_S can adsorb. Key examples of this include porous metal oxides^10^ and amine-functionalized mesoporous silicas.^9,10^

In addition to the types of sorbents described above, a variety of composite materials involving metal oxides for H_2_S sorption and gas sensing have also been developed.^15−18^ Many of the synthetic approaches used to develop these materials involve the deposition of a metal salt onto a suitable support material, which is then calcined to generate metal oxide. For example, Orojlou et al. deposited NiO, CuO, and CoO onto commercially available P25 TiO_2_ nanoparticles to create highly effective sorbents for H_2_S.^15^ A similar approach was also employed by Doan et al., who investigated the preparation of a mesoporous ZnO/CuO nanocomposite for H_2_S sensing by depositing copper chloride onto a zeolitic imidazolate framework, followed by subsequent annealing.^16^ Other approaches have utilized polymers to create nanocomposite materials. Nassar et al. created films formed from acrylamide/ethylene vinyl acetate copolymers and CdO nanoparticles for use as potential sorbents in oil and gas drilling operations.^17^ Chemical adsorption of H_2_S onto the material occurred during breakthrough experiments onto the metal oxide and also through acid–base reactions with the amine groups of the polymer.^17^

Approaches that have studied creating porous structures from metal oxides have had success in improving their H_2_S adsorption capacity.^19−21^ For example, Tran synthesized porous ZnO and Ni-doped ZnO to create reactive materials and composites that showed desulfurization performance that was several times greater than commercial ZnO.^20^ Similarly, Liu et al. utilized polyethylene glycol as a structure-directing agent to create mesoporous nanocrystalline iron oxides that showed a substantially larger H_2_S removal capacity than commercially available iron oxide.^19^ Although these studies were highly effective at creating metal oxides that showed high efficacies for desulfurization, the high temperatures required (>500 °C) in their syntheses make them more challenging and potentially environmentally harmful to carry out at larger scales. Furthermore, residual material from the compounds used to direct the morphology can be difficult to remove from the surface, which could impact the desulfurization performance.^19^ Because of this, further research into scalable syntheses of highly reactive metal oxide surfaces that do not possess these considerations could greatly enhance the field. The development of suitable low-temperature synthetic routes for effective desulfurizers could greatly facilitate the deployment of materials for protection and deodorization and inhibit key detrimental processes, such as the S poisoning of catalysts for hydrogen production.^22^

In this paper, we investigate whether the particle morphology and surface area of the Cu_4_(OH)_6_SO_4_ and CuO particles that precipitate from solution when NaOH_(aq)_ is added to CuSO_4(aq)_ at room temperature can be controlled and whether it is possible to substantially enhance the ability of the particles to adsorb H_2_S as a result of varying these properties. Herein, we show using STEM that varying the mol ratio of NaOH/CuSO_4_ can have a profound effect on particle morphology, which can greatly affect the performance of the materials when acting as sorbents for H_2_S in breakthrough experiments. The materials reported herein show desulfurization performance that is over an order of magnitude greater than commercially available CuO nanoparticles when applied onto polymer foam to make filters, indicating that this relatively straightforward synthetic procedure is highly effective for creating sorbents for H_2_S.

To our knowledge, this is the first report studying a nanocomposite material that utilizes Cu_4_(OH)_6_SO_4_ in addition to CuO. Furthermore, we are not aware of any systematic investigations that study how the particle morphology that is created when NaOH_(aq)_ is added to CuSO_4(aq)_ affects the H_2_S adsorption behavior. Analysis of our XPS data indicates that Cu_4_(OH)_6_SO_4_, in addition to CuO, is responsible for chemically adsorbing H_2_S, which contributes to its effectiveness.

## 2 Materials and Methods

### 2.1 Materials

Copper sulfate pentahydrate and sodium hydroxide were purchased from Fisher Scientific. Copper oxide nanopowder (<50 nm particle size) and zinc oxide nanopowder (<50 nm particle size) were obtained from Merck Life Sciences Ltd., in addition to activated charcoal. γFe_2_O_3_ nanoparticles (20 nm) were purchased from US Research Nanomaterials.

### 2.2 CuO/Cu_4_(OH)_6_SO_4_ Synthesis

A 0.5 M NaOH_(aq)_ solution was added dropwise to a stirring solution of 1 M CuSO_4(aq)_ over approximately 4 h until the suspension became highly viscous and dark blue in color. The mixture was left to oxidize overnight, whereby it darkened and changed to a black/gray color. Different volumes of NaOH_(aq)_ were added to typically 75 mL of CuSO_4(aq)_ to investigate what effect altering the mol ratio (mol NaOH/mol CuSO_4_) had on the material properties. Studies into varying the concentration of NaOH_(aq)_ and its addition time were also carried out. However, these were not observed to have a marked effect on the composition of CuO/Cu_4_(OH)_6_SO_4_ or its surface area. Following the oxidation, the mixture was then centrifuged at 4122*g* for 1 h to collect the solid. The recovered solid was resuspended in water and then centrifuged once again under the same conditions. This process was repeated two more times. The resulting slurry was dried overnight at 60 °C to afford a black/gray-colored solid.

### 2.3 Characterization

XRD was carried out using a Bruker D8 Discover diffractometer equipped with a nonmonochromatic Cu Kα X-ray source. Data were recorded over a 2θ range of 10–70° using a step time of 0.5 s and an increment of 0.02° per step. Scans were analyzed using the instrument’s Diffrac.EVA software. Percentages of the CuO and Cu_4_(OH)_6_SO_4_ present in the samples were calculated using Rietveld refinement using TOPAS software in the 20–70° 2θ range [*R*_exp_, *R*_wp_, *R*_p_, and GOF values for selected samples are shown in the Supporting Information (Table S1)]. N_2_ sorption analysis at 77 K was performed on a Quantachrome Instruments Nova 2000 multi-station nitrogen adsorption analyzer. Samples weighing approximately 300–500 mg were degassed under vacuum for at least 2 h ahead of analysis. The BET equation was fitted in the 0.201–0.281 *P*/*P*_0_ range. Scanning transmission electron microscopy (STEM) imaging was carried out using a Thermo Fisher Scientific Talos F200X instrument equipped with an EDS detector. Scanning electron microscopy (SEM) was performed using a Hitachi S4800 tabletop microscope. Diffuse reflectance UV–vis measurements were performed using an Agilent Technologies Cary Series UV–vis spectrophotometer in the range of 400–800 nm. The data interval was 1.0 nm, and the time per step was 0.2 s.

### 2.4 H_2_S Breakthrough Experiments

#### 2.4.1 Filter Preparation

1.00 g portions of sorbent material were dispersed in 15 mL of isopropanol, and the resulting suspensions stirred for 2 days at room temperature. The suspensions were then pipetted onto pieces of polyurethane foam (2.5 mm thickness, 25 mm diameter) that were placed on a Buchner funnel with a flask attached. The flask was attached to a vacuum pump in a manner similar to that used in filtration. A vacuum was then applied during the coating to draw the suspension through the foam. A number of different sorbent loadings were applied to the foam in order to better evaluate the efficacies of the different mixtures.

The coated pieces were then sandwiched in between discs of Gore-Tex (3.0 μm pore size) and plastic (Cryovac MF540, 100 μm thickness) that were the same diameter as the foam pieces. The foam pieces were attached to these materials by applying thin strips of adhesive around the outer edges of the discs. Finally, the discs were attached by applying the adhesive strips in the same manner onto the other side of the Gore-Tex and then sticking the assembly over a 13 mm hole made into a plastic film [(75 μm thickness), composed of 5-ply ethylene vinyl acetate/ethylene vinyl acetate/poly vinylidene dichloride/ethylene vinyl acetate/ethylene vinyl acetate]. The attachment to the plastic was required in order to securely clamp the filters into position during breakthrough testing.

#### 2.4.2 Breakthrough Testing

A 25 ppm H_2_S flow in CH_4_ (20%) and N_2_ (79.9975%) was allowed to flow through the 13 mm holes present in the Gore-Tex side of the filters at a rate of 1.2 L/min. During the experiments, gas passes out of the exposed sides of the foam and not the top plastic cover layer. Tests were completed when the concentration of H_2_S passing through the samples reached 2.0 ppm. Sample material was recovered from the foam pieces that had been exposed to H_2_S for XPS characterization. The foam pieces were sonicated in isopropanol to remove the sorbent that had been exposed to the test gas. The recovered sorbent was collected using centrifugation and then dried at 70 °C.

## 3 Results and Discussion

### 3.1 Characterization of the CuO/Cu_4_(OH)_6_SO_4_ Sorbent

It has been proposed that CuO is formed from the addition of NaOH_(aq)_ to CuSO_4(aq)_ through an intermediate basic copper sulfate, brochantite, Cu_4_(OH)_6_SO_4_, by the following precipitation reactions^23,24^
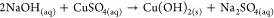
1
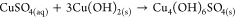
2

**Figure 1:**
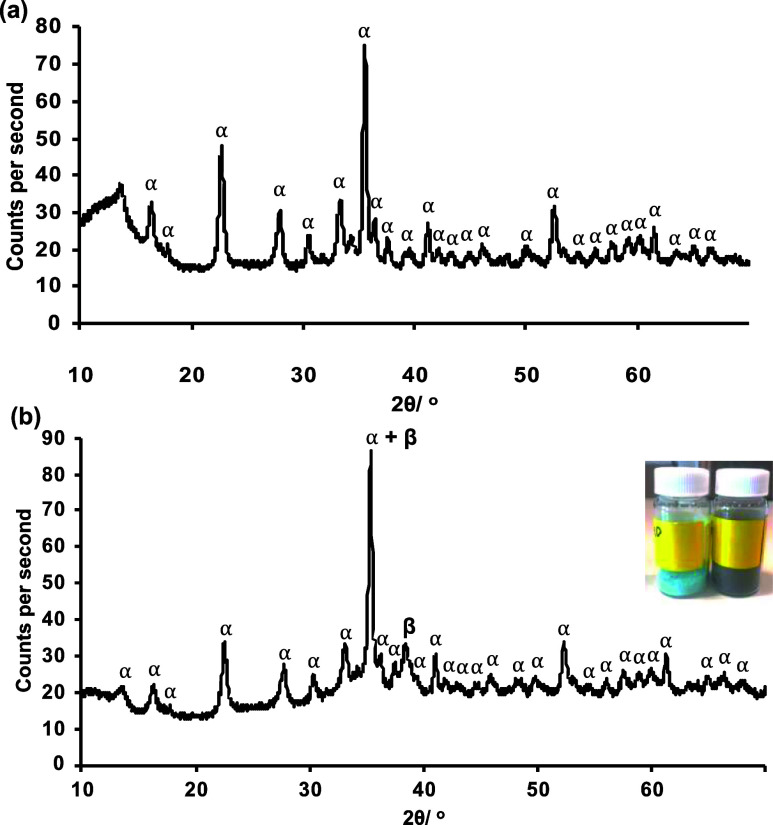
XRD diffractograms of a sample containing pure brochantite (a) and a sample containing 16.50% tenorite (b) (Table 1 entry 2) α = Cu_4_(OH)_6_SO_4_ and β = CuO. The inset of (b) shows vials containing brochantite (left) and a brochantite/tenorite mixture (right).

Further addition of NaOH_(aq)_ to the reaction mixture lowers the pH so that it is now thermodynamically favorable for brochantite to dehydrate and form tenorite, CuO, in the suspension.^18^ It was observed using XRD that it was possible to form mixtures solely containing CuO and Cu_4_(OH)_6_SO_4_ where the mol ratio of NaOH/CuSO_4_ was increased to a value of at least 1.39 (Table 1) without forming any other hydrated basic copper sulfate phases (langite, posnjakite, etc.).^23^ Below this ratio, only peaks ascribed to brochantite (Figure 1a) were observed using XRD, suggesting that the pH was too low for tenorite to form. Increasing the amount of NaOH_(aq)_ in the reaction mixture further led to the production of tenorite, as observed by the diffraction peak at a 2θ value of 39° (Figure 1b). The reflection at the 2θ value of 39° was observed at a higher intensity for samples prepared using a greater mol ratio of NaOH/CuSO_4_, in addition to new reflections at higher 2θ values, which were also ascribed to the tenorite phase (Figure S2). Rietveld refinement was performed on the XRD data to study the relative proportions of the two phases observed. It was observed that the percentage of CuO formed was highly sensitive to the moles of sodium hydroxide added to CuSO_4(aq)_ when the mol ratio of the reagents was increased to values greater than 1.39. This is not unsurprising since Marani et al.^23^ have reported that there is a sharp phase boundary between brochantite and tenorite at approximately pH 8. In line with this, increasing the mol ratio from 1.39 to 1.61 was observed to increase the amount of tenorite in the mixture from approximately 9 to 47% (Table 1). Increasing the mol ratio further was observed to create samples solely comprising tenorite.

**Table 1:** Relative Amounts of Tenorite and Brochantite Observed in Samples Produced by the Reaction of NaOH_(aq)_ with CuSO_4(aq)_ and Their Respective Surface Areas^a^

mol ratio NaOH/CuSO_4_	% CuO	% Cu_4_(OH)_6_SO_4_	specific surface area/m^2^ g^–^^1^
1.39	9.1	90.9	78.3
1.45	16.5	83.5	71.4
1.51	22.5	77.5	59.5
1.61	46.9	53.1	54.8

a Amounts of both phases are calculated using Rietveld refinement, details of which are shown in the Supporting Information (Table S1).

**Figure 2:**
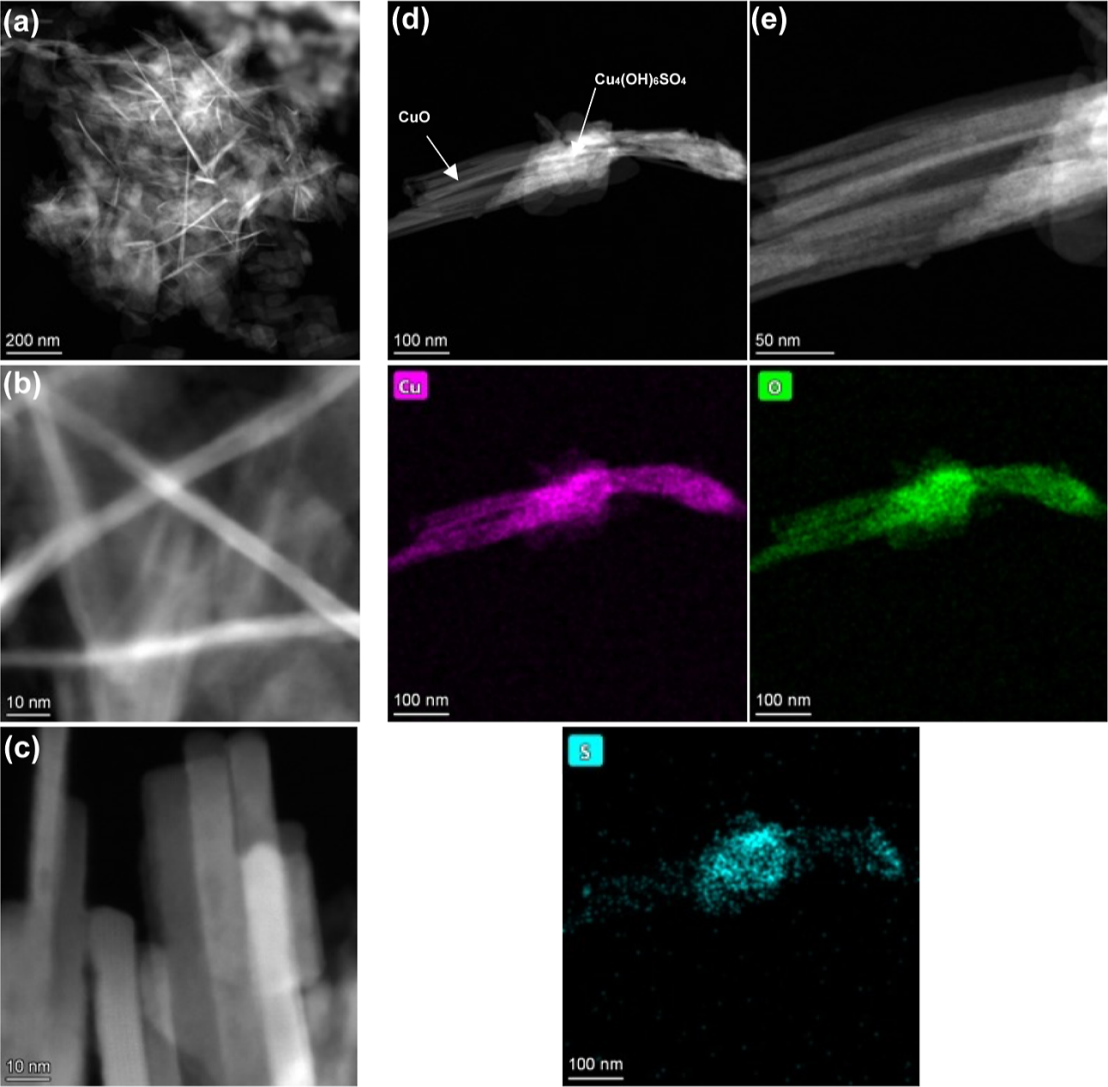
STEM images of the CuO/Cu_4_(OH)_6_SO_4_ particles with 14.17% CuO content (a,b), 46.89% CuO content (c), and a particle with a central brochantite region that also showed tenorite lamellar (d,e). Corresponding EDS maps were taken at the same magnification as (d). The map in pink corresponds to Cu, while the maps in green and turquoise correspond to O and S, respectively.

In addition, samples containing brochantite/tenorite mixtures were a dark color as opposed to pure brochantite samples that were emerald green, and the precursor, copper sulfate pentahydrate, which was blue (Figure 1b inset and Figure S1b). Diffuse reflectance UV–vis spectroscopy was also employed to study the optical properties of the different materials, where it was observed that the colored compounds showed distinct bands at 528 and 469 nm (Figure S1a) for brochantite and copper sulfate pentahydrate, respectively. As anticipated, the dark-colored mixture did not show any bands within the visible region.

BET analysis on the Cu_4_(OH)_6_SO_4_/CuO mixtures shown in Table 1 indicated that the samples formed using lower mol ratios of NaOH/CuSO_4_ had larger surface areas. Samples formed from pure brochantite had substantially lower surface areas than those shown in Table 1 (ca. 46 m^2^ g^–1^), suggesting that the CuO that had formed in the reaction mixture possessed a far higher surface area. STEM was employed to investigate this in more detail. Analysis of the particle morphologies showed that the samples were highly polydisperse and were a mixture of irregularly shaped particles (Figure S3) and needle-like particles, some of which were less than 10 nm in thickness (Figure 2a,b). It was observed that samples comprised of less tenorite (<20% CuO) contained more of the needle-like particles, such as those observed in Figure 2, suggesting that it was this morphology that was responsible for the higher surface areas measured during the BET analysis. Unfortunately, it was not possible to distinguish between the types of particles using EDS since they were highly agglomerated, thus making it impossible to ascertain chemical identities because of the large interaction volume of the electron beam relative to that of the nanostructures. However, related reports studying the alkaline precipitation of Cu(II) compounds in the presence of sulfate have ascribed this wire-like morphology to be characteristic of the tenorite phase.^23^ By comparison, STEM performed on samples that only contained brochantite showed that the particles were various sizes and shapes and contained far fewer particles that could be described as needle-like structures (Figure S4). Further investigations into the morphology of the particles using the STEM revealed the presence of larger lamellar structures formed in the lower surface area higher CuO samples (Figure 2c–e). Particle agglomeration made it difficult to assign the identities of the different structures using EDS. However, mapping revealed that lamellae contained more Cu and O and less S. Figure 2d,e shows an example of a lamellae structure agglomerated with a brochantite-rich area, wherein we can observe from the elemental mapping that the sheets of the lamellae contain more of the tenorite phase.

### 3.2 Interaction of CuO/Cu_4_(OH)_6_SO_4_ Sorbents with H_2_S and Breakthrough Behavior

**Scheme 1:**
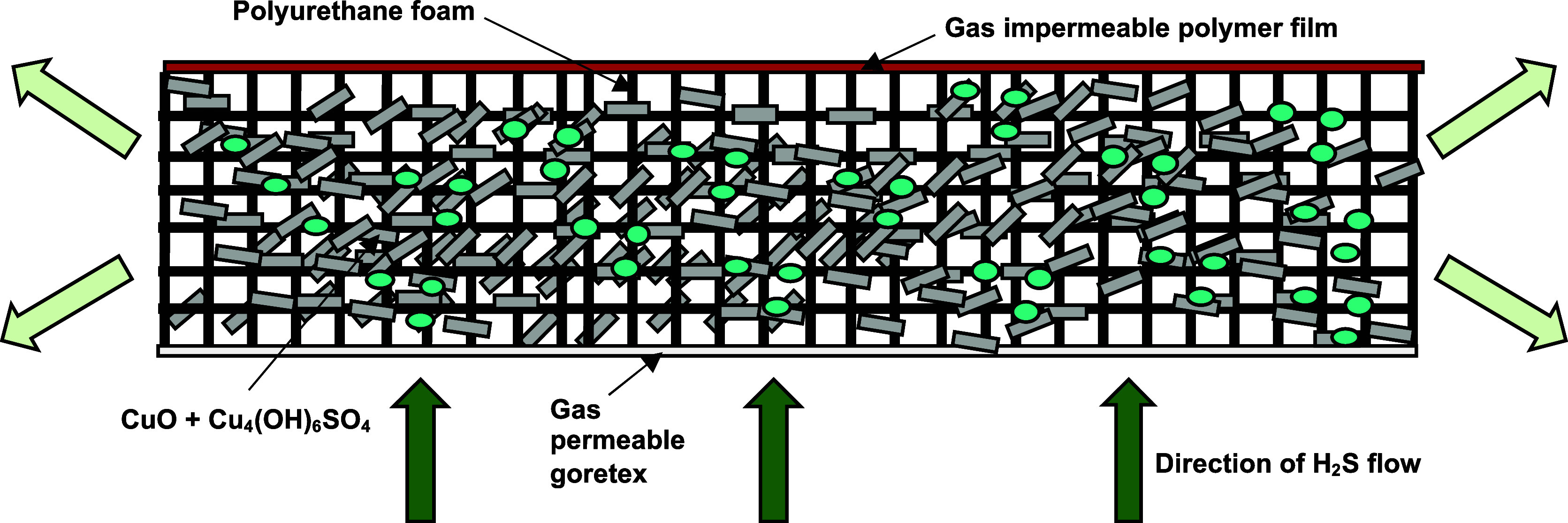
Design of the Filters Used in the H_2_S Breakthrough Experiments The arrows that show the gas stream exiting the filter are a lighter green color than those entering to display the desulfurization of the mixture by the sorbent.

**Figure 3:**
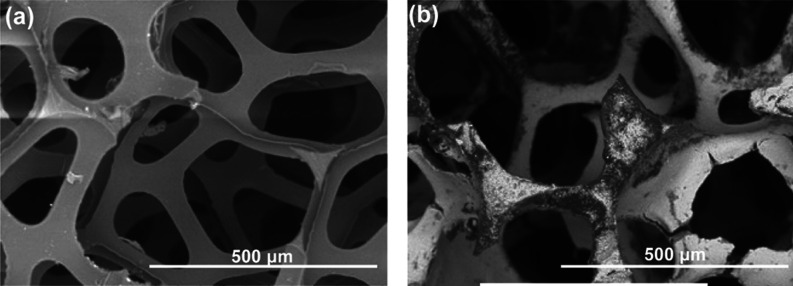
Backscattered electron images in the SEM of polyurethane foam (a) and the foam coated with the Cu_4_(OH)_6_SO_4_/CuO sorbent (b).

Experiments to study the efficacy of the mixtures to adsorb H_2_S were conducted by applying the mixtures onto polyurethane foam and then using the coated foam to prepare filters for a 25 ppm H_2_S gas stream. A porous hydrophobic membrane was attached to one side of the foam, and a gas-impermeable plastic was attached to the other side. During the tests, gas flows out of the side edges of the foam and not through the plastic, as indicated in Scheme 1. SEM analysis of the coated pieces showed that the particles were loaded onto the foam as agglomerates of various sizes, with some webbing observed between the pores (Figure 3). However, plenty of pores remained open to facilitate gas flow. Images of the uncoated and coated foam pieces are displayed in Figure S1c,d to show the coverage of the foam by the coating.

**Figure 4:**
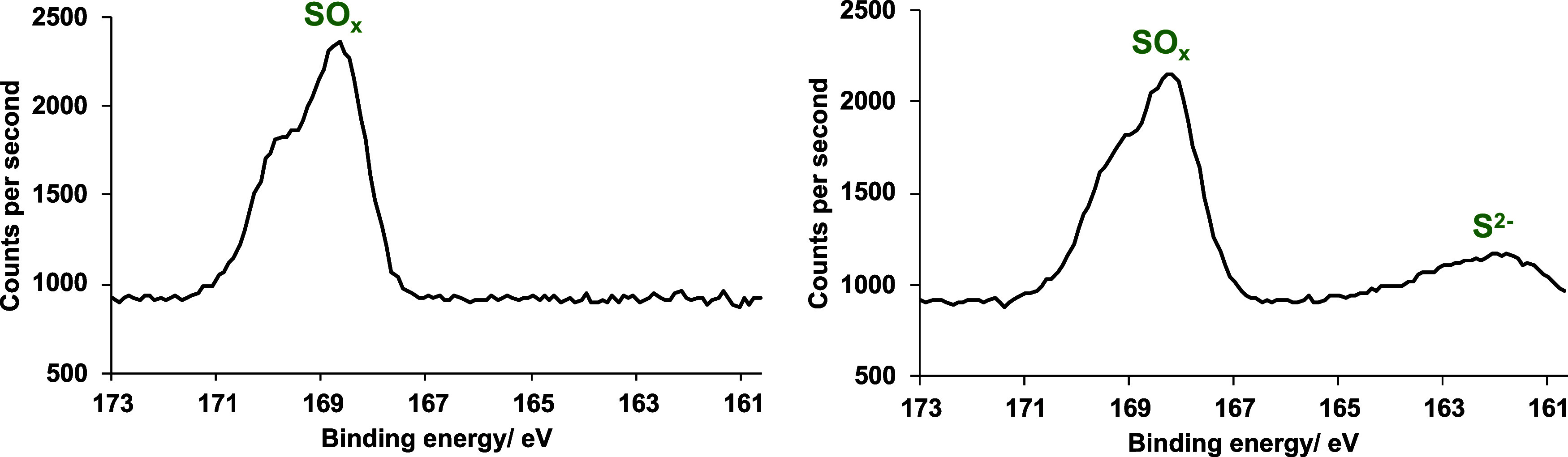
High-resolution XPS spectra of the S 2p region for pure brochantite samples before (a) and after (b) exposure to H_2_S.

XPS data showed that both Cu_4_(OH)_6_SO_4_ and Cu_4_(OH)_6_SO_4_/CuO mixtures were reactive toward H_2_S. Material recovered from the coated foam showed the anticipated S 2p photoelectron peaks at approximately 168.6 eV ascribed to the sulfate moiety of brochantite prior to exposure to the test gas.^12^ Following exposure, peaks ascribed to sulfides were also observed in the XPS spectra at about 162.5 eV, indicating that areas of the samples had reacted with H_2_S (Figures 4 and S6).^12^ In addition, two S 2s peaks were observed following treatment with H_2_S, providing additional evidence that sulfides had been formed (Figures S6 and S7). The peak at 230.2 eV is ascribed to the sulfate moiety of brochantite, whereas the peak at 223.5 eV is ascribed to sulfide species.^25^ XPS survey spectra of Cu_4_(OH)_6_SO_4_ and a Cu_4_(OH)_6_SO_4_/CuO mixture are also displayed in the Supporting Information (Figure S5). XRD carried out on Cu_4_(OH)_6_SO_4_ and a Cu_4_(OH)_6_SO_4_/CuO mixture did not show any peaks that could be ascribed to CuS, which could suggest that this material has a low degree of crystallinity. However, since this reaction involves only atoms in the uppermost surface layers, it might be more plausible that the material generated might be below the instrument’s limit of detection.

It has been proposed that sulfidation of CuO by hydrogen sulfide proceeds through dissociative adsorption of H_2_S followed by a redox reaction between the surface Cu^2+^ ions and S^2–^, whereby Cu^2+^ is first reduced to Cu^+^.^12^ The S^2–^ is oxidized to sulfate compounds, which remain adsorbed to the surface layer. Once all of the surface Cu(II) has been reduced, it is then believed that further dissociative adsorption of H_2_S leads to an exchange mechanism between O^2–^ and S^2–^, which causes the replacement of the oxide ions by the sulfide ions and forms water as a byproduct. Since Cu_4_(OH)_6_SO_4_ contains Cu(II), it is plausible that it could react similarly, first through reduction of Cu(II) to Cu(I) and then through exchanges of hydroxide and sulfide ions, which would lead to water being formed as a byproduct after combining with the surface adsorbed H atoms.

Filters were created using polymer foam substrates impregnated with Cu_4_(OH)_6_SO_4_ and Cu_4_(OH)_6_SO_4_/CuO mixtures to study the efficacies of the different sorbents in H_2_S breakthrough studies. During the experiments, H_2_S (25 ppm) was flowed through the coated foam, and the concentration of H_2_S that passed through the substrates was monitored. Tests were finished when the concentration of H_2_S leaving the substrates was recorded to be 2.0 ppm. Figure S9 shows the breakthrough behavior of H_2_S through the sorbent containing 15.50% CuO. As expected, the loading dependence on breakthrough behavior is quite marked, with lower-loaded filters showing quite a rapid breakthrough time, which is probably due to poor coverage of the substrate by the sorbent. Shallower slopes are observed in the more linear portions of the curves for samples containing higher sorbent loadings (>73 mg). This indicates that the larger amounts of sorbent present react with more H_2_S molecules, causing a reduced breakthrough of the gas.

**Figure 5:**
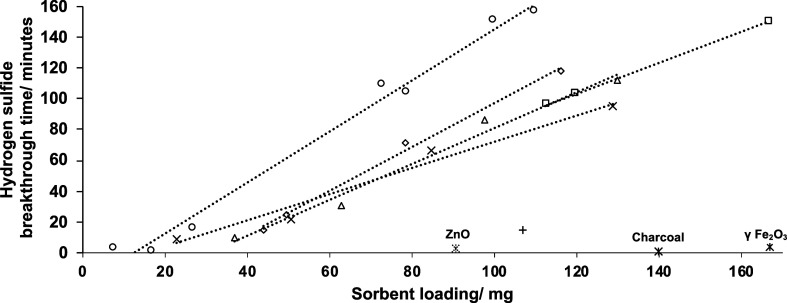
Plots comparing filters prepared using sorbents with different CuO contents: 0% CuO (squares), 9.13% CuO (diamonds), 16.50% (circles), 22.51% CuO (triangles), and 46.89% CuO (crosses). The plus sign represents a filter prepared using commercially available CuO. Also included in the graph are breakthrough times of filters prepared with commercially available materials that have been reported to adsorb H_2_S.

Evaluating the efficacy of the different sorbents, it was observed that filters prepared solely using Cu_4_(OH)_6_SO_4_ had considerable efficacy but not as great as the higher surface area Cu_4_(OH)_6_SO_4_/CuO sorbents that contained the rod-like particles (Figure 5). Interestingly, it was observed that the best-performing mixture (15.50% CuO) did not possess the largest surface area (Table 1). This could suggest that a balance between surface area and loading exists where there is a threshold above which it is more desirable to have more CuO as opposed to having a mixture of largely Cu_4_(OH)_6_SO_4_ with a small amount of higher surface area CuO. However, as the CuO content increases further and larger particles form with lower surface area-to-volume ratios, the performance drops, which leads to a substantial loss of efficacy. Despite that, Cu_4_(OH)_6_SO_4_/CuO mixtures with 57.40% CuO still show substantially higher breakthrough times than commercially available CuO nanoparticles that had a size range of <50 nm (Figure 5). BET analysis of the commercially available CuO nanomaterials revealed that they only had a surface area of 16.2 m^2^ g^–1^, while TEM showed that the material was largely composed of particles that were more spherical in shape (Figure S10). This provides further support that the greater efficacy observed for the Cu_4_(OH)_6_SO_4_/CuO mixtures is due to the higher surface area-to-volume ratio of the rod-shaped particles, which provide a greater number of sites for H_2_S adsorption.

Further to this, the efficacy of the Cu_4_(OH)_6_SO_4_/CuO mixtures was also studied against commercially available metal oxide nanoparticles and activated charcoal, both of which have been shown to be reactive toward H_2_S.^10,11^ It was observed that the gas broke through filters prepared using these materials in several minutes, even at loadings toward the top end of our measurement range. BET analysis revealed that the surface areas of the metal oxides were within the range observed for the particles in the Cu_4_(OH)_6_SO_4_/CuO mixtures (Table S2), while the surface area of the charcoal was higher as anticipated. This suggests that the Cu_4_(OH)_6_SO_4_ and CuO particles synthesized using this method are far more reactive toward H_2_S under these conditions and are thus better suited for use in filters exposed to this flow of H_2_S.

The methodology developed in this study shows that it is possible to create high-performance sorbent materials for H_2_S by using an aqueous route using relatively inexpensive, commercially available precursors at ambient conditions. The simplicity and control that this method affords have the potential to significantly advance the field since the synthesis can be conducted using basic laboratory equipment and relatively benign chemicals, allowing it to be readily upscaled for applications demanding large amounts of sorbent material. Examples of cases that could utilize this level of demand could include the production of sorbent material for filters fitted to protective clothing used where there are high levels of H_2_S release, such as in oil or gas wells,^10,11^ or in areas where harmful concentrations of the pollutant are released from wastewater treatment facilities.^26^ The facile nature of its production also means that this sorbent could be utilized in filters in disposable devices in the medical sector, thus improving the quality of patients’ lives and care.

## 4 Conclusions

In this paper, we have shown that the efficacy of sorbents for H_2_S adsorption can be substantially increased through optimizing synthetic conditions to create particles that show higher surface area-to-volume ratios. The Cu_4_(OH)_6_SO_4_/CuO mixtures are produced at ambient temperature from readily available, relatively benign chemicals in an aqueous environment, thus making their fabrication very accessible for widespread production. This attractive synthetic route could be useful for producing sorbent material for a variety of applications where H_2_S poses a risk or a problem. Key examples of this could include the use of this material to stop the inhibition of catalysts used in hydrogen production through sulfur poisoning^22^ or the application of the sorbent onto suitable filter media in the healthcare sector.

This study shows that it is possible to control the relative amounts of Cu_4_(OH)_6_SO_4_ and CuO that are formed through simply varying the mol ratio of NaOH/CuSO_4_ and the amount of needle-like CuO particles within the mixture. STEM shows that these particles can be as thin as 10 nm in thickness and as long as several hundred nanometers in length. As such, this high aspect ratio affords the particles a far greater number of surface sites for H_2_S to associate with, relative to spherical particles, for example, which results in far greater performance. This work demonstrates that it is possible to form these structures in solution without the addition of surfactants, which have previously been utilized in related reports to facilitate particle growth in particular directions.^27^ However, the formation of these higher surface area morphologies can only be realized at certain stoichiometries; otherwise, larger copper oxide particles form with lower surface area-to-volume ratios, which show lower efficacy toward adsorbing H_2_S. Previous studies have not reported enhanced adsorption behavior of H_2_S on needle-like copper oxide particles relative to particles with other shapes.^28^ However, their investigation studied particles with far greater thicknesses, which were toward the micron scale,^28^ and thus possessed substantially lower surface area-to-volume ratios than what we have observed in our study.

In addition, this study also shows that H_2_S can chemically adsorb onto brochantite in a manner similar to that onto copper oxide, thus making it an effective sorbent for the gas. The basic copper sulfate (0% CuO) was found to show greater efficacy toward adsorbing H_2_S relative to off-the-shelf copper oxide nanoparticles, which helps explain the high performance of the CuO/Cu_4_(OH)_6_SO_4_ mixtures. To the best of our knowledge, the H_2_S-adsorbing ability of Cu_4_(OH)_6_SO_4_ has not previously been reported. In summary, our investigation has shown that the particles in the CuO/Cu_4_(OH)_6_SO_4_ mixtures synthesized using this simple, wet chemical method are highly reactive toward H_2_S. This reactivity is over an order of magnitude greater than other metal oxide nanoparticles that have been previously studied for use as sorbents for H_2_S removal.

The high efficacy of this sorbent mixture has been utilized in this investigation when applied to porous polymer foams to create effective filters for the malodorous gas, which could find application in the medical sector as filters or in protective clothing articles that are worn in environments where people are exposed to high levels of H_2_S.^10,11^

## Supporting Information Available

Additional XRD and TEM data, XPS results, and BET data of several commercial nanoparticles (PDF)
