# Supplementary material for: Development of CuO/Cu4(OH)6SO4 Nanoparticle Mixtures to Optimize the H2S Adsorption
Source: ACS Appl Eng Mater. 2024 Jan 22;2(2):305–12. doi: 10.1021/acsaenm.3c00575 (PMC10897877; doi:10.1021/acsaenm.3c00575)
Supplement: Supplementary file 1 — em3c00575_si_001.pdf [file em3c00575_si_001.pdf]

Supporting Information

Development of CuO/ $Cu_4(OH)_6SO_4$ nanoparticle mixtures to optimise the H_2S adsorption

*Donald Hill[1], Yubiao Niu[2], Henry Apsey [1], Omotoke Olonisakin[1], Richard Palmer[2] and Shirin Alexander[1]**

[1]Energy Safety Research Institute (ESRI), Faculty of Science and Engineering, Swansea University Bay Campus, Fabian Way, Swansea SA1 8EN, UK

[2] Nanomaterials Lab, Faculty of Science and Engineering, Swansea University, Bay Campus, Fabian Way, Swansea, SA1 8EN, UK

*S.Alexander@swansea.ac.uk

Table S1 R factors obtained from Rietveld refinement of CuO/ $Cu_4(OH)_6SO_4$ powder XRD patterns.

**Mol ratio NaOH: $CuSO_4$**	**% CuO**	**% $Cu_4(OH)_6SO_4$**	**R_{exp}**	**R_{wp}**	**R_p**	**GOF**
1.39	9.13	90.87	1.61	4.06	2.94	2.52
1.45	16.5	83.5	2.16	3.87	2.84	1.79
1.51	22.51	77.49	2.26	3.53	2.67	1.56
1.61	46.89	53.11	1.27	4.09	2.99	3.22

(a)

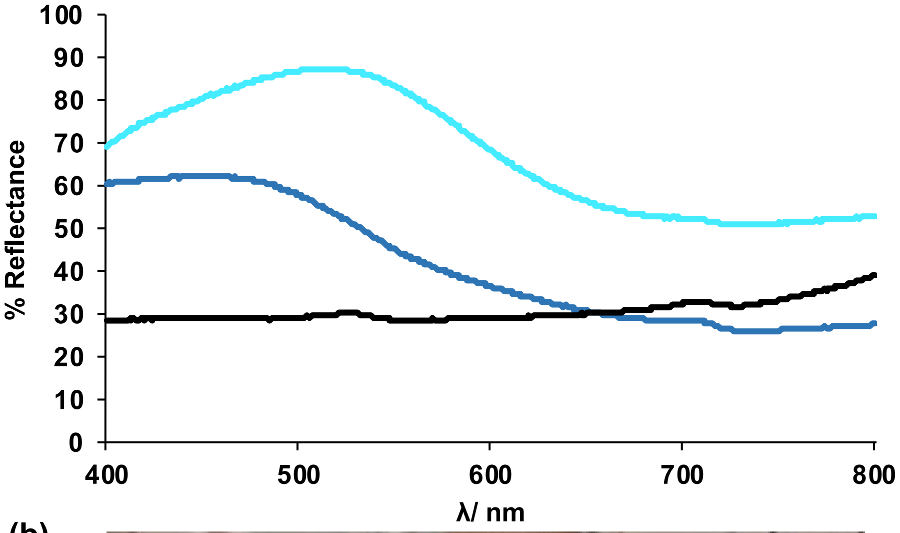

(b)

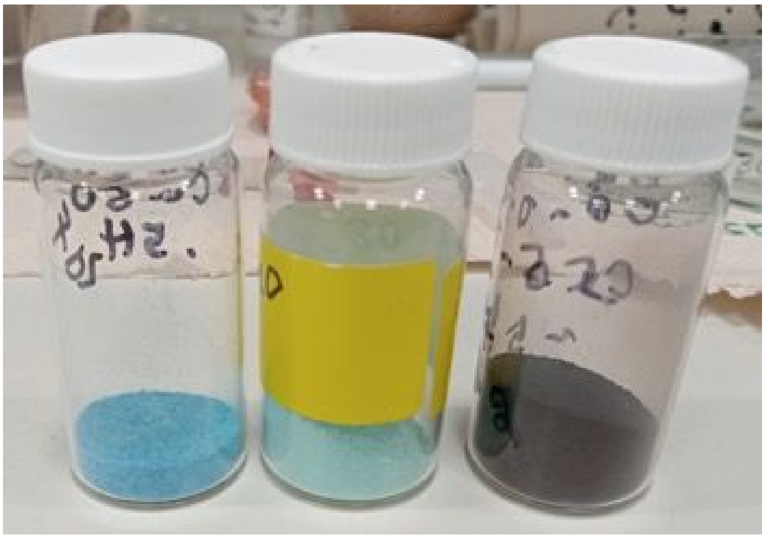

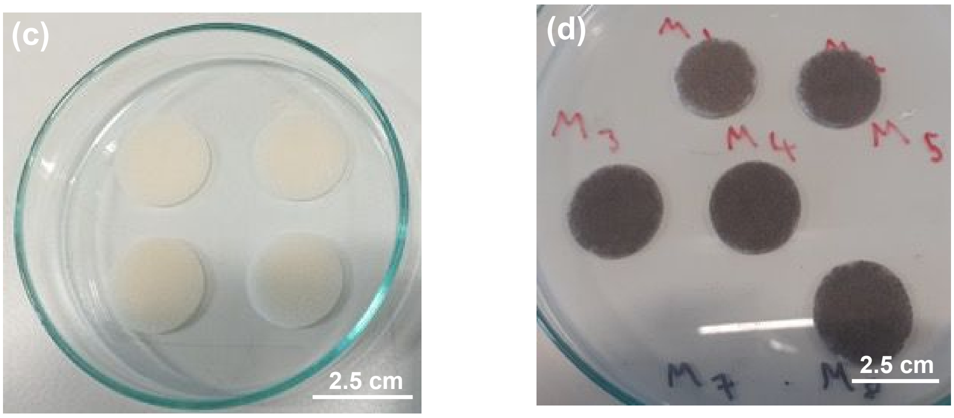

Figure S1. (a) Diffuse Reflectance UV-vis spectra of brochantite (turquoise series, top), copper sulfate pentahydrate (blue series, middle) and a $Cu_4(OH)_6SO_4$/ CuO mixture containing 46.9 % CuO (black series, bottom). (b) Vials containing copper sulphate pentahydrate (left), pure $Cu_4(OH)_6SO_4$ (middle) and the $Cu_4(OH)_6SO_4$/ CuO mixture containing 46.9 % CuO (right). Pieces of the uncoated and coated foam are shown in (c) and (d) respectively.

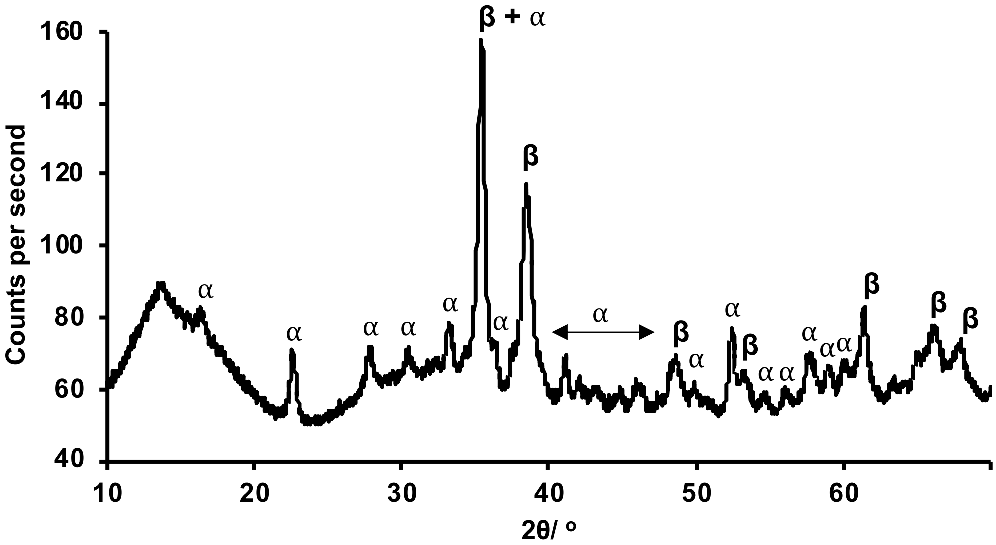

Figure S2. XRD diffractogram of the sample containing 46.89 % CuO. α = $Cu_4(OH)_6SO_4$ and β = CuO.

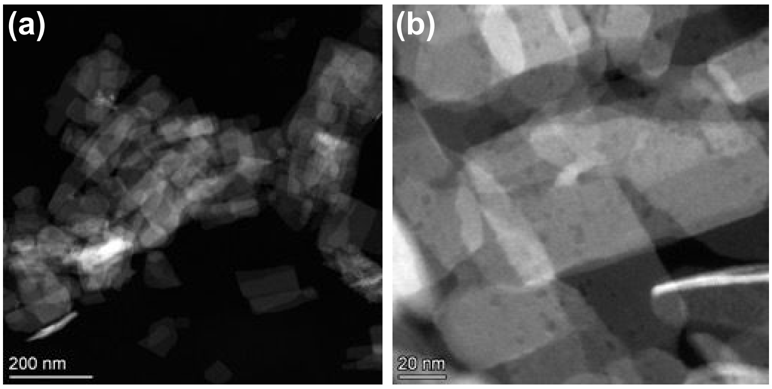

Figure S3. TEM images taken at lower (a) and higher (b) magnification of the $CuO/ Cu_4(OH)_6SO_4$ particles with 14.17 % CuO content.

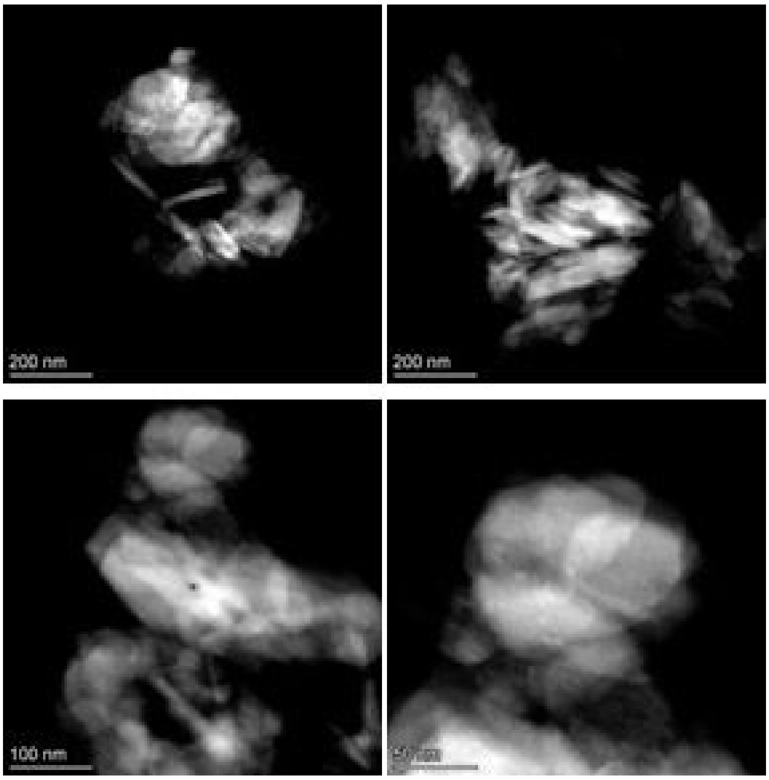

Figure S4. TEM images of brochantite particles.

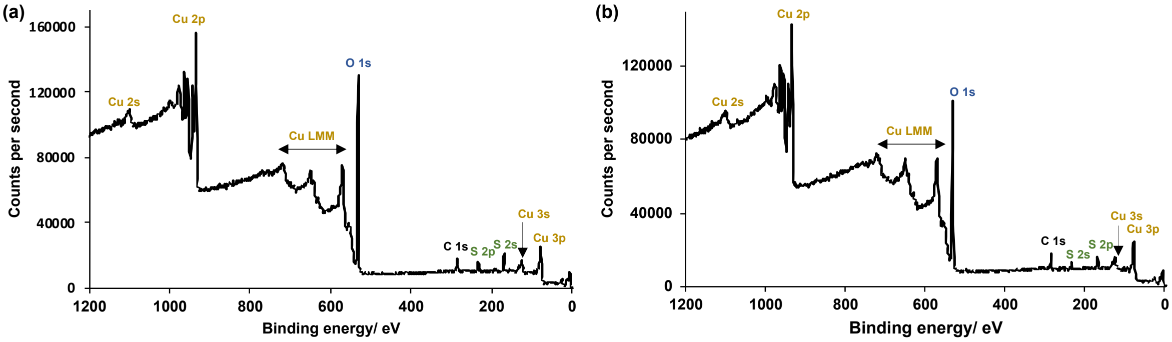

Figure S5. XPS survey spectra of brochantite (a) and of a $Cu_4(OH)_6SO_4$/ CuO mixture containing 25.18 % CuO (b).

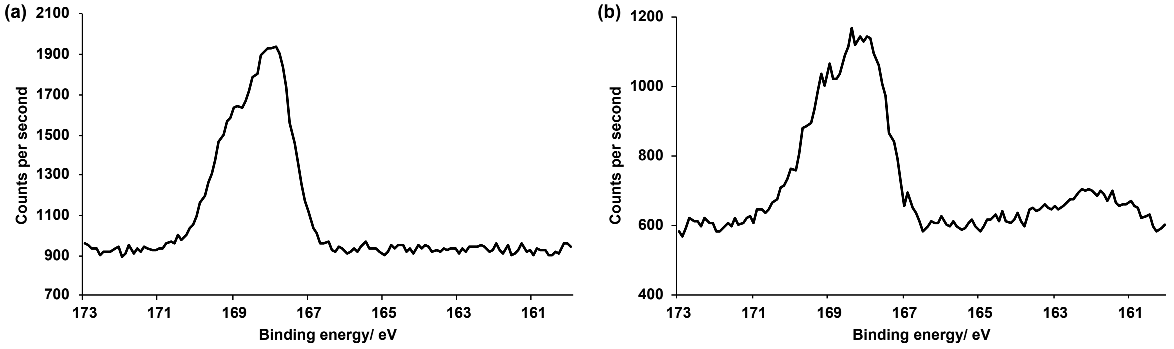

Figure S6. High resolution XPS spectra of the S 2p region of a sample containing 25.18 % CuO before (a) and after (b) exposure to H_2S.

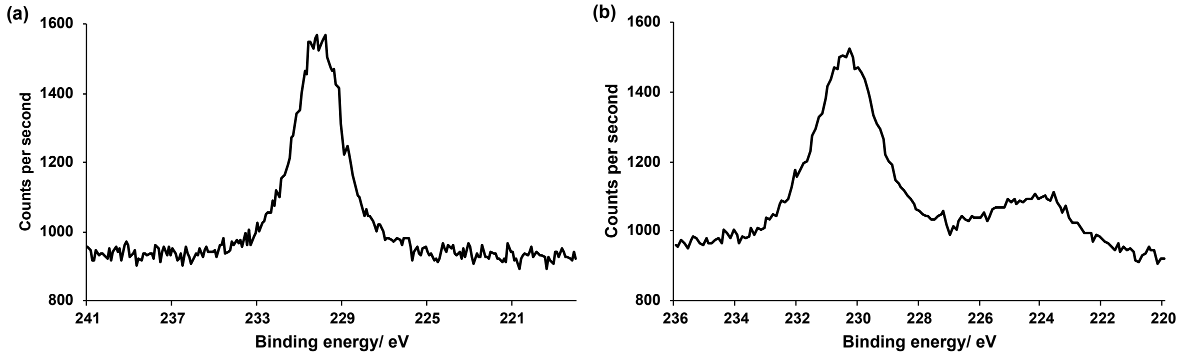

Figure S7. High resolution XPS spectra of the S 2s region of a brochantite sample before (a) and after (b) exposure of H_2S.

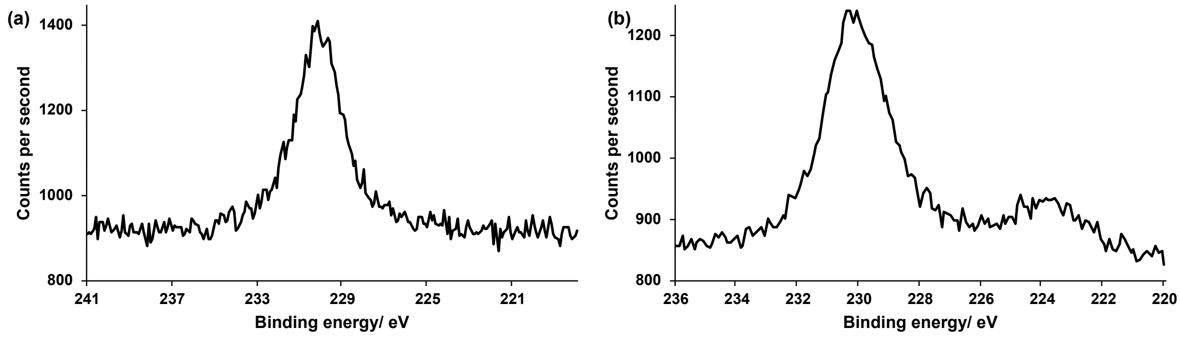

Figure S8. High resolution XPS spectra of the S 2s region of a sample containing 25.18 % CuO before (a) and after (b) exposure of H_2S.

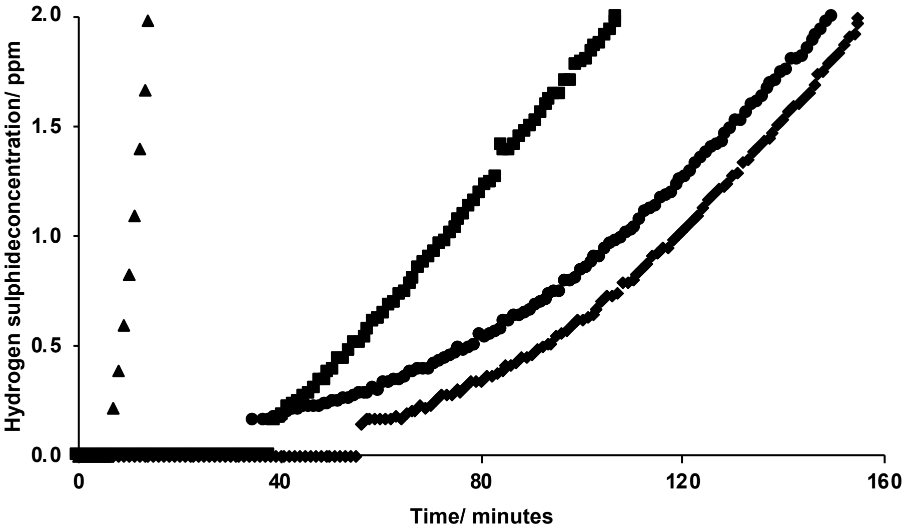

Figure S9. Curves showing the breakthrough behaviour of H_2S through filters prepared using different loadings of a sorbent containing 15.50% CuO: 27 mg (triangles), 73 mg (squares), 100 mg (circles) and 110 mg (diamonds).

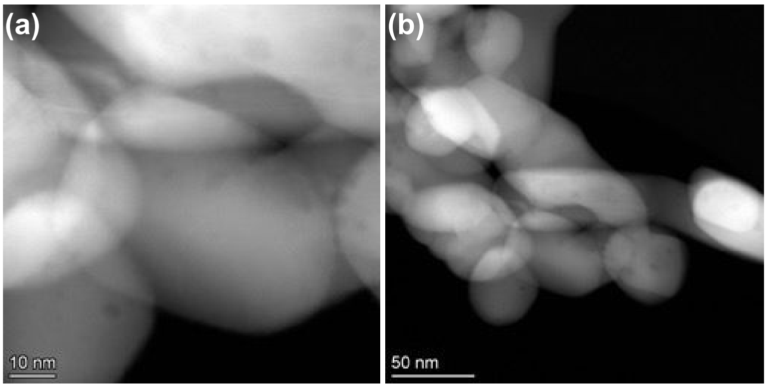

Figure S10. TEM images of commercially available 50 nm CuO taken at higher (a) and lower (b) magnification.

Table S2. Surface areas of other materials calculated from BET measurements.

Material	Surface area/ m^2g^{-1}
Zinc oxide (< 50 nm)	35
γ Fe_2O_3 (20 nm)	76
Copper Oxide (50 nm)	16
Activated Charcoal	978
